# The ASACUSA antihydrogen and hydrogen program: results and prospects

**DOI:** 10.1098/rsta.2017.0273

**Published:** 2018-02-19

**Authors:** C. Malbrunot, C. Amsler, S. Arguedas Cuendis, H. Breuker, P. Dupre, M. Fleck, H. Higaki, Y. Kanai, B. Kolbinger, N. Kuroda, M. Leali, V. Mäckel, V. Mascagna, O. Massiczek, Y. Matsuda, Y. Nagata, M. C. Simon, H. Spitzer, M. Tajima, S. Ulmer, L. Venturelli, E. Widmann, M. Wiesinger, Y. Yamazaki, J. Zmeskal

**Affiliations:** 1 Experimental Physics Department, CERN, Genève 23, 1211, Switzerland; 2 Stefan-Meyer-Institut für Subatomare Physik, Österreichische Akademie der Wissenschaften, Boltzmanngasse 3, 1090 Wien, Austria; 3 Ulmer Fundamental Symmetries Laboratory, RIKEN, Wako, Saitama 351-0198, Japan; 4 Graduate School of Advanced Sciences of Matter, Hiroshima University, Hiroshima 739-8530, Japan; 5 Nishina Center for Accelerator-Based Science, RIKEN, Wako, Saitama 351-0198, Japan; 6 Institute of Physics, The University of Tokyo, Komaba, Meguro-ku, Tokyo 153-8902, Japan; 7 Dipartimento di Ingegneria dell’Informazione, Università di Brescia, Brescia 25133, Italy; 8 Istituto Nazionale di Fisica Nucleare, Sez. di Pavia, 27100 Pavia, Italy; 9 Department of Physics, Tokyo University of Science, Shinjuku, Tokyo 162-8601, Japan

**Keywords:** antihydrogen, hyperfine spectroscopy, Standard Model Extension, atomic beam

## Abstract

The goal of the ASACUSA-CUSP collaboration at the Antiproton Decelerator of CERN is to measure the ground-state hyperfine splitting of antihydrogen using an atomic spectroscopy beamline. A milestone was achieved in 2012 through the detection of 80 antihydrogen atoms 2.7 m away from their production region. This was the first observation of ‘cold’ antihydrogen in a magnetic field free region. In parallel to the progress on the antihydrogen production, the spectroscopy beamline was tested with a source of hydrogen. This led to a measurement at a relative precision of 2.7×10^−9^ which constitutes the most precise measurement of the hydrogen hyperfine splitting in a beam. Further measurements with an upgraded hydrogen apparatus are motivated by CPT and Lorentz violation tests in the framework of the Standard Model Extension. Unlike for hydrogen, the antihydrogen experiment is complicated by the difficulty of synthesizing enough cold antiatoms in the ground state. The first antihydrogen quantum states scan at the entrance of the spectroscopy apparatus was realized in 2016 and is presented here. The prospects for a ppm measurement are also discussed.

This article is part of the Theo Murphy meeting issue ‘Antiproton physics in the ELENA era’.

## 1. Introduction

Since the first detection of relativistic antihydrogen atoms more than 20 years ago at LEAR (CERN) [1] and later at Fermilab [2], the field of antihydrogen research rapidly took momentum with the start of the Antiproton Decelerator (AD) at CERN in 2000. The first detection of low energy antihydrogen was reported in 2002 [3,4] followed by the first magnetic trapping of antihydrogen in 2010 [5] which enabled the first measurements on trapped antihydrogen atoms in the following years. Given the magnetic moment of antihydrogen in the ground state, trapping requires typical temperatures smaller than approximately 0.5 K which is challenging since the adopted formation mechanism requires the interaction of trapped antiproton and positron clouds. The ASACUSA-CUSP collaboration proposed in 2005 a measurement of the ground-state hyperfine splitting of antihydrogen using a beam method allowing a relaxed constraint, of about two orders of magnitude, on the temperature of the antihydrogen atoms available for measurements [6].^1^

The race towards producing large amount of cold antihydrogen atoms is motivated by the appealing prospects for CPT (combination of charge conjugation, parity transformation and time reversal) symmetry tests. The measured atomic transitions on hydrogen, one of the best studied atomic systems, constitute a precise comparison ground for antihydrogen. A direct consequence of the CPT theorem [7–10] is that antihydrogen and hydrogen should have the exact same spectrum. Measuring atomic transitions in antihydrogen with high precision therefore promises one of the most stringent tests of CPT symmetry. The motivation behind testing such a cornerstone of quantum field theory is manifold. The baryon asymmetry in the universe reflected by the notable absence of primordial antimatter remains to-date unexplained. Additionally, quantum field theory, although extremely successful, yet fails to include the gravitational force.

Tests of CPT symmetry have been and are being performed in several different physical systems. In the leptonic sector, the symmetry has been tested by comparison of the electron and positron as well as the charged muon g-factors [11,12]. The charge-to-mass ratio of the antiproton currently provides the most precise CPT test in the baryon sector [13] to which can be added the recently reported comparison at the ppb level of the magnetic moment of the proton and the antiproton [14,15] and the extraction of the antiproton mass through spectroscopic measurement in antiprotonic helium [16]. In mesons, the famous neutral kaon mass comparison [17] provides one the most stringent CPT test to date. Finally, in nuclei recent measurements were reported in ALICE of the charge-to-mass ratio of anti-helium nuclei and anti-deuterons [18]. The so far only accessible atomic system purely consisting of antimatter is antihydrogen, for which the first optical transitions between the 1S and 2S states were observed paving a path to precise measurements [19]. The ground-state hyperfine splitting has been very recently measured with a relative precision of 4×10^−4^ [20]. Relative precisions are commonly used to compare the sensitivity of experiments. In the context of CPT symmetry tests, the Standard Model Extension (SME) [21,22] sets a framework in which experiments searching for CPT and Lorentz symmetry violations can be compared to each other. In this framework, CPT violation arises from the inclusion of all possible effective operators for Lorentz violation to the standard model Lagrangian. A Lorentz-violating term in the Lagrangian of the SME is constructed from a tensor coefficient contracted with a conventional tensor operator. The coefficients act as Lorentz-violating background fields. For most low-energy systems, the absolute energy scale probed by the experiments defines their sensitivity to those fields. For antihydrogen [23,24], the 1S–2S transition and the ground-state hyperfine splitting, to cite only the most precisely measured transitions in hydrogen, are sensitive to different coefficients of the SME fields and can therefore provide different constraints to potential new physics.

The antihydrogen trap experiments’ early focus was dedicated to the 1S–2S transition^2^^,^^3^ which was measured for hydrogen in a trap with a relative precision of 2×10^−12^ [25]. An almost three orders of magnitude more precise value was achieved in a beam in 2011 [26]. The ASACUSA-CUSP collaboration intends to measure the ground-state hyperfine splitting of antihydrogen in a beam. The same transition in hydrogen was measured in a beam (before the new ASACUSA-CUSP measurement, see §2b) with a precision of 5×10^−8^ [27,28]; a four orders of magnitude more precise value was achieved in a maser in the 1970s [29–34].

The advantage of the beam setup for the measurement of the hyperfine splitting of antihydrogen is that, on top of having a larger temperature acceptance than traps, it allows a measurement in a nearly magnetic field-free region. Indeed the hyperfine splitting being a magnetic phenomenon, the measurable transitions between the different hyperfine states are very sensitive to magnetic field gradients. Thus, the technique in principle allows for better resolution, accuracy and precision on the hyperfine splitting than a measurement in a trap. The challenges however lie in forming a polarized beam of ground-state antihydrogen atoms.

In the first place a focused beam of antihydrogen should be produced to compensate for the loss of solid angle due to the distance between the production and the detection points. In the current design of the ASACUSA-CUSP antihydrogen apparatus [35], the antihydrogen detector sees a solid angle of 5×10^−4^ sr. Even the most efficient production of antihydrogen, which nowadays converts approximately 30% of the roughly 10^5^ trapped antiprotons, would only achieve a couple of antihydrogen at the detector per trial without a beam formation. Secondly, the beam should be polarized. In a trap the polarization is automatic as the untrappable states (called high-field seekers) annihilate on the surrounding electrodes right after their production and only the trappable states (two low-field seeker states with total angular momentum quantum number *F* and magnetic quantum number *M*_*F*_ equal to (*F*,*M*_*F*_)=(1,0) and (*F*,*M*_*F*_)=(1,1), the latter being (*F*,*M*_*F*_)=(1,−1) for antihydrogen) remain in the trap. A third challenge in a beam method relates to the quantum states of the antihydrogen atoms. Three-body recombination, which is the dominating production mechanism in the ASACUSA-CUSP experiment [36], mostly produces antihydrogen in highly excited states [37]. They spontaneously decay to the ground state within nanosecond (for low principal quantum number *n*) to millisecond (for e.g. circular states of *n*∼30) time scales. The temperature acceptance of the ASACUSA-CUSP apparatus is limited to roughly 50 K, which translates into velocities of approximately 1000 m s^−1^. Such velocities in a beam do not allow enough time for decays from high-lying *n*-states (*n*>25 [38]) to happen before the atoms have reached the spectroscopy apparatus. By contrast, atoms can remain in a trap for tens of minutes. Therefore, in a beam, the production of ground state, or nearly ground state, atoms needs to be enhanced or stimulated de-excitation mechanisms need to be implemented.

We have underlined here the experimental challenges specific to the ground-state hyperfine splitting measurement of antihydrogen in a beam and we will discuss in §3 how they are being addressed by the ASACUSA-CUSP collaboration. In the next section, we will describe the results yielded by a test apparatus used on hydrogen and motivate further measurements in hydrogen in the context of the SME.

## 2. Hyperfine splitting measurement

### (a) Theoretical background

The hyperfine splitting in (anti)hydrogen arises from the interaction of the magnetic moments of the electron (positron) and proton (antiproton). To first order, it is proportional to the product of those. The magnetic moment of the antiproton is now known to the ppb level [14]. At the 10^−5^ level [39], corrections to the first-order calculation of the hyperfine splitting introduce contributions due to the magnetic and electric form factors of the antiproton which are to date unknown. Therefore, given the current knowledge on the magnetic moment of the antiproton, the ground-state hyperfine splitting of antihydrogen is sensitive at the 30 ppm level to the structure of the antiproton. This strongly motivates, in addition to the prospect for more sensitive CPT tests, further experiments beyond the currently achieved relative precision of 4×10^−4^ in a trap.

**Figure 1.**
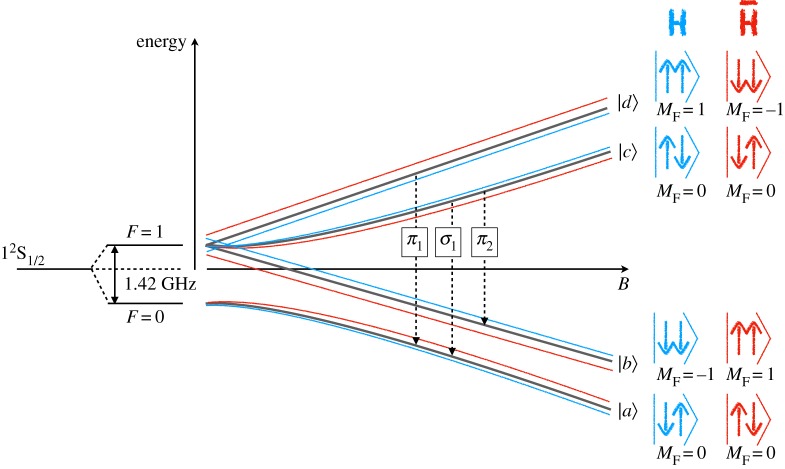
Illustrative figure of the Breit–Rabi diagram and the potential effect of the SME’s CPT and Lorentz violating fields on the hyperfine splitting of hydrogen and antihydrogen. The sign and magnitude of the shift affecting each state within hydrogen (or antihydrogen) are dependent on the magnitude of the *g* and *H* coefficients in equation (2.2) as well as the *c* and *a* coefficients mentioned in the text [24,40]. Here, we have assumed that the effect on hydrogen and antihydrogen is opposite and of different, non-zero, amplitude which at least implies that *g*≠*H*. (Online version in colour.)

In the presence of an external magnetic field, the hyperfine levels are further split due to the additional interaction term proportional to *μB* (where *μ* is the hydrogen magnetic moment and *B* the magnetic field) in the Hamiltonian. Figure 1 illustrates how the *F*=1 triplet state’s degeneracy is lifted in a magnetic field and the dependence of the states on the magnetic field’s amplitude. In this figure, the |*d*〉 and |*c*〉 states refer to the low-field seeking and |*b*〉 and |*a*〉 to the high-field seeking states mentioned above. Three transitions between low field seeking and high field seeking states are then possible. The *π*_1_ and *π*_2_ (for which Δ*M*=±1) as well as the *σ*_1_ (Δ*M*=0) transitions allow for the determination of the zero-field transition which can be compared to theoretical calculations (to the 10^−5^ level as noted above) or to other experimental measurements. The zero field value can be extracted through extrapolations by measuring the *π*_1_ or *σ*_1_ transitions at different external magnetic field and using the Breit–Rabi formula that relates the energy of the states to the external magnetic field value [41]. Alternatively one can measure two transitions at the same magnetic field to directly compute the zero-field value. In the presence of Lorentz and CPT violating effects, those two methods could lead to different results. One should note that the *σ* transition at zero field is not sensitive to SME fields because the shift of the hyperfine transitions is proportional to Δ*M* (see equation (2.2)). In general, computations which cancel the effect of the linear Zeeman shift also cancel the effect of Lorentz and CPT violating terms. This is the case of the difference between the *π*_1_ and *π*_2_ transitions which does not lead to any constraints on SME coefficients. Figure 1 illustrates the potential effect of SME fields on hydrogen and antihydrogen hyperfine structures. The magnitude and the sign of the effect for hydrogen and antihydrogen are unknown and depend on the relative strength of CPT-odd and CPT-even terms. In order to determine those, measurements on both matter and antimatter are necessary and should be realized at the same time and at the same location.

In hydrogen additional constraints on SME coefficients can be obtained by measuring sidereal variations of the hyperfine splitting which could be caused by the change of the magnetic field orientation (due to the rotation of the Earth) with respect to the background fields responsible for Lorentz violation. Similar constraints could be obtained in antihydrogen if the rate of antiatoms would permit such fast measurements. SME coefficients measured in the laboratory frame can be expressed in terms of coefficients in the Sun-centred frame (in which the Lorentz breaking background fields are assumed to be constant) as [24]
2.1
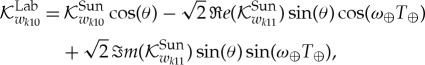
where *ω*_⊕_=2*π*/23  56 is the Earth’s rotation frequency, *T*_⊕_ is the sidereal time and *θ* is the angle between the applied magnetic field and the Earth’s rotational axis. The symbol 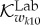 denotes any of the coefficients *g*^0*B*^, *H*^0*B*^, *g*^1*B*^, *H*^1*B*^ which are non-relativistic spherical coefficients for Lorentz violation expressed in the laboratory frame. For clarity, we have omitted here the superscript *NR* on every coefficient but it should be noted that all SME coefficients mentioned in this paper are non-relativistic. The 0 and 1 superscripts refer to the spin weight [40] and the *w* subscript stands for electron or proton. The index *k* represents the mass dimension of the coefficient. Here, we restrict to *k*≤4 given that the coefficients are suppressed by (*αm*)^2*k*^. The *g* coefficients control CPT-odd effects, while the *H* ones control CPT-even effects. Each specific coefficient governs a physically distinct violation of Lorentz symmetry. Two additional coefficients, *c* and *a*, are involved in the shift of the hyperfine states. However, they shift all hyperfine sub-levels of a principal state by the same amount and are therefore not discussed here, as they are not accessible by direct observation of the hyperfine splitting.

In the laboratory frame the shift of a hyperfine transition is given by
2.2
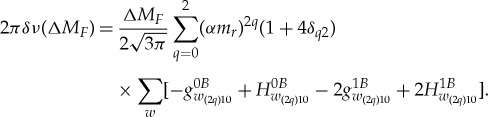
Here, *k*=2*q* and therefore *q* spans from 0 to 2. Since *w* = e,p and there are four types of non-relativistic coefficients, we count 3×2×4=24 independent coefficients contributing to the frequency shift of the *π* transition in the laboratory frame. Each laboratory-frame coefficient being associated with three independent coefficients in the Sun-centred frame (equation (2.1)), there are a total of 24×3=72 independent SME coefficients in the Sun-centred frame.

The measurement of the hyperfine transition using a maser reached an absolute precision of mHz [42–44]. Sidereal measurements at the same precision led to the constraint of 48 of the coefficients mentioned above (the ones embedded in 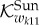) setting bounds at the level of 2×10^−27^ GeV [44]. The beam technique described in the next section can reach a precision of the order of a hertz and therefore cannot provide better constraints than the maser measurement on sidereal coefficients. However, the remaining 24 unconstrained coefficients (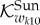) can be probed by changing the field orientation in the laboratory.

We have so far ignored contributions due to the rotation of the Earth around the Sun. If one considers this additional boosted and rotated frame, additional oscillations in the signal could be observed. Performing measurements at different times of the year would enable constraining additional coefficients which are to-date unprobed (but are however suppressed with respect to the ones listed above [24]).

### (b) Latest results on hydrogen

The measurement of the *σ*_1_ transition to an unprecedented precision in a beam was recently reported in [45]. The spectroscopy apparatus (a resonant cavity in which the hyperfine transition is driven and a superconducting sextupole magnet to select the spin state) designed for the ASACUSA-CUSP antihydrogen experiment (see §3a) was used for this measurement.

The *σ*_1_ transition, having a quadratic dependency on the external static magnetic field *B*, is less sensitive to the inhomogeneity of the field and could therefore be measured using a single pair of Helmholtz coils providing a field of a few gauss in a direction perpendicular to the beam and parallel to the radiofrequency (RF) field, the latter being necessary to drive the *σ* transition, and an homogeneity of *σ*_*B*_/*B*∼1% at the cavity. An uncertainty of a few hertz was reached (2.7 ppb relative precision) and no significant signs for systematic errors have been encountered at this level of precision. However, as mentioned in §2a, *σ*_1_ is insensitive to Lorentz and CPT violating SME fields and a new measurement campaign was started to measure with a similar precision the *π*_1_ transition. At 10 gauss the *σ*_1_ and *π*_1_ transitions differ by approximately 14 MHz. The cavity used is resonant at 1420 MHz with a bandwidth of 12 MHz allowing the measurement of both transitions.

**Figure 2.**
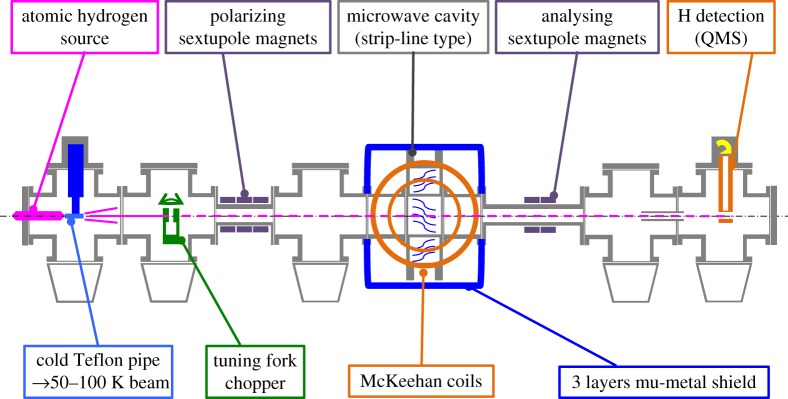
Sketch of the hydrogen experiment setup to measure the *π*_1_ transition.

We report here the first observation of the *π*_1_ transition in a beam. This measurement required an additional set of correction coils and a new magnetic shielding design which combined provided a field homogeneity better than *σ*_*B*_/*B*∼0.1%. The cavity is rotated by 45^°^ around the hydrogen beam axis to allow both the *σ* and *π* transitions to be driven (for the latter one, the external magnetic field needs to be perpendicular to the RF field). Figure 2 shows the experimental setup used for this measurement. The cavity and the two pairs of Helmholtz coils (in a so-called McKeehan configuration [46]) are enclosed in the magnetic shielding. The cavity is identical to the one previously used in the *σ*_1_ determination. Its ‘strip-line’ design leads to a lineshape with a double-dip structure (see [45] for more details on the lineshape’s structure). The superconducting sextupole, being used in the antihydrogen experiment detailed in §3, was replaced by sets of permanent magnets with a smaller diameter but a similar integrated gradient in order to allow both hydrogen and antihydrogen experiments to be operated independently.

**Figure 3.**
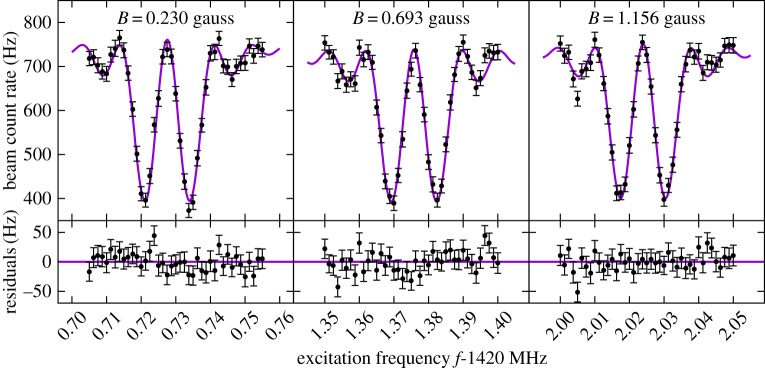
*π*_1_ transitions observed at different external magnetic field amplitudes. 41 data points are taken over a scan range of 50 kHz. The lineshape is fitted to extract the central frequency with approximately 60 Hz precision. Residuals are shown in the bottom plots. (Online version in colour.)

Figure 3 shows the *π*_1_ transition measured at three different fields. Since the homogeneity of the external field allows the resolution of the double-dip structure, the precision of this method is dominated by the interaction time of the atoms with the microwave field. A velocity of 1000 m s^−1^ leads to a linewidth of 12 kHz. With an acquisition time of 40 min, we achieved a precision on the central frequency of 60 Hz, which splits the observed linewidth by a factor of 200. A few hertz precision can be reached by measuring both *π*_1_ and *σ*_1_ transitions within a one week long measurement campaign [47].

### (c) Outlook

This result shows that new constraints on (or determination of!) SME coefficients can be made in the near future. Measurements of the *π*_1_ transition at opposite B-field directions using the *σ*_1_ transition (which is insensitive to SME fields) as a reference will be performed to access the 24 unconstrained coefficients mentioned in §2a. Comparison of the zero-field frequency obtained using the extrapolation method on the *π*_1_ transition and the combination of the *π*_1_ and *σ*_1_ transitions will provide additional constraints. In a second step, further measurements at different times of the year will enable assessing other un-constrained coefficients. It is also worth noting that the hydrogen experiment is located at the same Earth’s latitude and longitude coordinates as our analogue antihydrogen experiment.

## 3. Antihydrogen measurement

As already mentioned, antihydrogen spectroscopy will further constrain the SME landscape in particular the CPT-odd terms. The recent measurement of the hyperfine splitting in hydrogen enabled an estimation of the number of antihydrogen needed to reach a ppm relative precision using the same technique. Few assumptions on the antihydrogen beam properties were made which included an estimation of atoms in excited states in the cavity. An important difference between the hydrogen and antihydrogen experiment is indeed that antihydrogen atoms are formed in highly excited states. A determination of the quantum state distribution of antihydrogen atoms at the entrance of the spectroscopy apparatus was therefore necessary. It was performed using an external field-ionizer (described below) upstream of the cavity to reject atoms in a high principal quantum number state. The result of the measurement done in the ASACUSA-CUSP antihydrogen apparatus is presented and discussed in §3b.

### (a) Status

**Figure 4.**
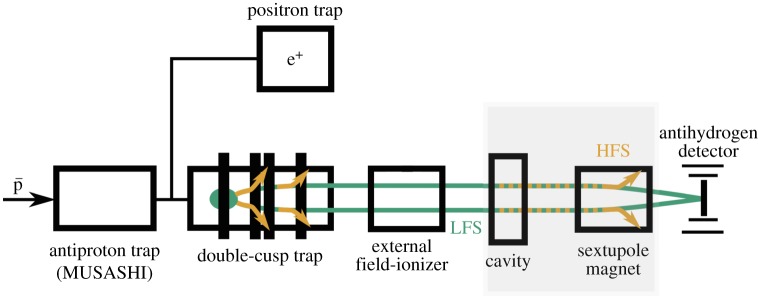
Sketch of the ASACUSA-CUSP antihydrogen setup. The spectroscopy apparatus used in the hydrogen *σ*_1_ measurement reported in [45] is highlighted by the grey box. For the quantum state measurements the highlighted components were removed, and the antihydrogen detector was placed directly downstream of the external field-ionizer. (Online version in colour.)

The first measurement of antihydrogen atoms in a field-free environment, 2.7 m away from the production region was reported by the ASACUSA-CUSP collaboration in 2014 [48]. Since then, efforts were concentrated on increasing the flux and characterizing beam properties in view of the spectroscopic measurements. For this latter purpose, a field-ionizer capable of ionizing states down to principle quantum number *n*∼14 [35] was added directly upstream of the cavity. Together with the field-ionizer internal to the CUSP and closer to the production region it provided a diagnostic on the states of the antihydrogen produced inside and exiting the CUSP. During the 2016 data-taking period, the antihydrogen detector seen in figure 4 was placed directly downstream of the field-ionizer. The detector consists of a central bismuth germanium oxide (BGO) calorimeter read out by multi-anode photomultipliers [49] (therefore additionally providing position resolution) and a two-layer hodoscope made of 32 scintillator bars each, read out on both sides by silicon photomultipliers [50]. The combination of the vertexing capability of the detector and the measurement of the energy deposit at the annihilation point provides a strong discriminating power between cosmic (the main background component) and antihydrogen signals. Figure 4 shows the ASACUSA-CUSP antihydrogen apparatus. Synthesis of antihydrogen is done in a double-cusp trap inside which multi-ring electrodes provide the electrostatic field necessary to trap the charged particles axially. The double-cusp trap provides a strong magnetic field gradient [51,52] which should enhance the polarizing and focusing effect on the exiting antihydrogen beam.

### (b) Measurement of the quantum state distribution

We present here the first quantum state distribution measurement of antihydrogen atoms in a low-magnetic field region. The analysis of the data was done using gradient boosted decision trees, a machine learning algorithm, which was trained on antiprotons extracted from the MUSASHI trap towards the detector. The annihilation pattern of those antiprotons is from the detector’s point of view identical to those of antihydrogen. About 4000 annihilations of antiprotons were recorded. Two-thirds of those were used to train the algorithm and the other third was used to test the algorithm and extract the signal efficiency. Additionally about 30 000 cosmic events were recorded and two-thirds used to train the algorithm on background recognition. The efficiency of the algorithm in detecting antiprotons is close to 80% while the rate of mis-identified antiproton events in the cosmic sample is less than 0.25% (the details of the analysis procedure will be published elsewhere [53]). The angle of the tracks (tracks are defined as originating from the BGO detector) recorded in the detector with respect to the horizontal axis and the energy deposited in the BGO detector turned out to be the most important features for discriminating annihilation events from cosmic background.

**Figure 5.**
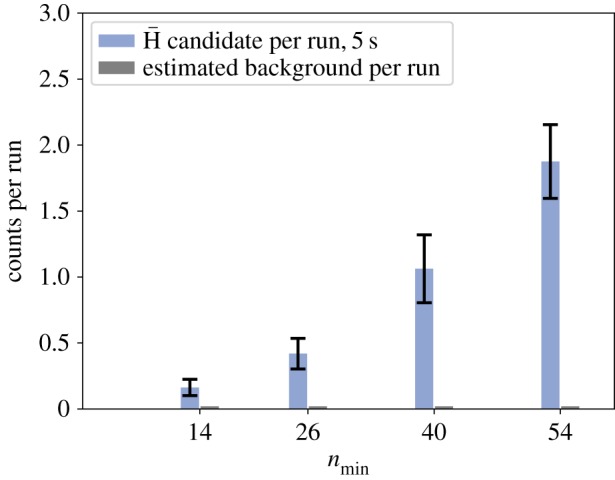
Measurement of the quantum state distribution of antihydrogen atoms at the entrance of the spectroscopy apparatus. The indicated quantum number *n*_min_ is the minimum principal quantum number ionized. The counts on the detector at that particular *n*_min_ therefore include all atoms having a principal quantum number *n*<*n*_min_. The counts are averaged over the first 5 s after the start of the mixing of antiprotons and positrons in the double-cusp trap. Poisson errors on the antihydrogen candidates are indicated. The errors on the background events are estimated by averaging the algorithm’s outcome to 500 randomly chosen training samples. They are too small to be seen on the graph. (Online version in colour.)

The algorithm was then used to identify antihydrogen events during mixing runs with four different settings of the external field-ionizer. The result of the analysis, normalized to a run, is shown in figure 5. The cosmic trigger rate in the detector is of the order of 1.6 Hz so that in a mixing run of 5 s, approximately eight cosmic events are expected leading to 0.02 fake antihydrogen events. The smallest number of antihydrogen events was recorded for the highest field-ionizer configuration (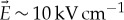) and averaged to about 0.16 events per run. Given the cosmic rejection, the background rate is more than eight times smaller. The antihydrogen atoms which are not ionized by the strongest field of the field-ionizer have a principal quantum number smaller than 14. From this state, the longest decay channel to the ground state is of the order of 100 μs. Assuming velocities of 1000 m s^−1^ (which is roughly the acceptance limit of the apparatus), antihydrogen atoms in those states would be in the ground state before they reach the cavity, apart from atoms decaying to the metastable state 2S. Within 43 mixing runs, 7 antihydrogen events with principal quantum number *n*<14 were recorded with a significance of 4.5*σ*.

### (c) Prospects

The first measurement of the antihydrogen quantum state distribution down to low quantum states confirms the higher proportion of high Rydberg states at the exit of the production trap. At this stage, the observed rate of low lying states is too small to reach the necessary number of antiatoms for a ppm measurement. Efforts are now concentrated on stimulating the de-excitation close to the production point as well as enhancing the production of ground-state antihydrogen. For that purpose, different mixing schemes are being developed and positron density and temperature are being optimized.

The Extra Low Energy Antiproton Ring ELENA [54] which is being commissioned at the AD, will provide, starting from 2021 for the majority of antihydrogen experiments, a lower beam energy and a higher beam availability which will be beneficial to the ASACUSA-CUSP experiment on three fronts: a higher number of low energy antiprotons, a round-the-clock antiproton availability which will avoid the daily time consuming beam-tuning through the ASACUSA RFQD and a separate beamline for the second ASACUSA activity, sparing the bi-annual disassembly and assembly of the entire apparatus and therefore allowing for necessary developments throughout the year.

## 4. Conclusions and outlook

We have presented the latest results of the hydrogen and antihydrogen experiments of the ASACUSA-CUSP collaboration. The recent hydrogen result followed by the first observation of the *π*_1_ transition opens the way to further measurements which will provide additional constraints on SME coefficients. In the antihydrogen experiment, first atoms with a low principal quantum number were detected at the entrance of the spectrometer. This result calls for a stimulated de-excitation in order to reach a rate of approximately 10 ground-state counts per run which would be compatible with a ppm measurement.

## Footnotes

1 ASACUSA proposal addendum, CERN/SPSC 2005-002, SPSC P-307 Add.1 (2005).

2 ALPHA proposal, CERN-SPSC-2005-006; SPSC-P-325 (2005).

3 ATRAP proposal, CERN-SPSC-97-8; SPSC-P-306 (1997).

## Data accessibility

This article has no additional data.
